# Male-killing endosymbionts: influence of environmental conditions on persistence of host metapopulation

**DOI:** 10.1186/1471-2148-8-243

**Published:** 2008-09-02

**Authors:** Dries Bonte, Thomas Hovestadt, Hans-Joachim Poethke

**Affiliations:** 1 Würzburg University, Field Station Fabrikschleichach, Glashuettenstrasse 5, D-96181, Rauhenebrach, Germany; 2 Terrestrial Ecology Unit, Department of Biology, Ghent University, K.L. Ledeganckstraat 35, BE-9000, Ghent, Belgium

## Abstract

**Background:**

Male killing endosymbionts manipulate their arthropod host reproduction by only allowing female embryos to develop into infected females and killing all male offspring. Because of the reproductive manipulation, we expect them to have an effect on the evolution of host dispersal rates. In addition, male killing endosymbionts are expected to approach fixation when fitness of infected individuals is larger than that of uninfected ones and when transmission from mother to offspring is nearly perfect. They then vanish as the host population crashes. High observed infection rates and among-population variation in natural systems can consequently not be explained if defense mechanisms are absent and when transmission efficiency is perfect.

**Results:**

By simulating the host-endosymbiont dynamics in an individual-based metapopulation model we show that male killing endosymbionts increase host dispersal rates. No fitness compensations were built into the model for male killing endosymbionts, but they spread as a group beneficial trait. Host and parasite populations face extinction under panmictic conditions, i.e. conditions that favor the evolution of high dispersal in hosts. On the other hand, deterministic 'curing' (only parasite goes extinct) can occur under conditions of low dispersal, e.g. under low environmental stochasticity and high dispersal mortality. However, high and stable infection rates can be maintained in metapopulations over a considerable spectrum of conditions favoring intermediate levels of dispersal in the host.

**Conclusion:**

Male killing endosymbionts without explicit fitness compensation spread as a group selected trait into a metapopulation. Emergent feedbacks through increased evolutionary stable dispersal rates provide an alternative explanation for both, the high male-killing endosymbiont infection rates and the high among-population variation in local infection rates reported for some natural systems.

## Background

Bacterial endosymbionts currently attain a lot of interest, because of their widespread occurrence in arthropod hosts in which they often manipulate reproduction [1,2]. They are predominantly vertically transmitted from mother to offspring, although the lack between phylogenies of host and endosymbionts indicates that horizontal transfer should be possible [1,2]. The genera *Wolbachia *[2,3] and *Rickettsia *[4] belong to the best studied endosymbionts. Reproductive manipulations by these endosymbionts comprise parthenogenesis (i.e. infected virgin females produce daughters), feminization (infected genetic males reproduce as females), cytoplasmatic incompatibility (CI; in its simplest form a cross between infected male and an uninfected female results in the death of embryos), and male killing (i.e. infected male embryos die and female embryos develop into infected females). In arthropods, they are considered as selfish elements that enhance their own transmission to the disadvantage of the rest of the genome [5-7] and strongly act as an evolutionary force on their hosts [8-13].

When the transmission efficiency from mother to offspring is (nearly) perfect [10,14-17], male-killing bacteria are expected to approach fixation when benefits for surviving daughters stem from the death of their male kin [5,18]. They then vanish as the host population crashes [5,17-20]. However, hosts do not reach extinction under these conditions if there is strong selection to prevent transmission of the parasite (e.g. through sexual selection of non-infected mates [3]) or when the phenotype is suppressed by host genes [11,21,22]. Turelli & Hoffmann [22] reported strong variation in transmission efficiency for CI-inducing endosymbionts between laboratory cultures and natural populations. Such variation can also be expected in male killing endosymbionts. Therefore, endosymbiont transmission rates may vary with temporal or spatial changes in the environment [17] and male killer prevalence will be reduced because some males always survive. It remains, however, an open question why many natural host populations that lack these defense mechanisms can persist in spite of high infection rates.

Male-killing bacteria are generally thought to attain low to intermediate prevalence in transient natural populations with only mild effects on host population sex ratio [20]. Strong heterogeneity in infection rates at intermediate spatial scales has been reported [16,20,23-26]. Charlat and colleagues [25] recently discovered that interactions with CI-inducing endosymbionts may explain natural variation in male killer prevalence in the butterfly *Hypolimnas bolina*. Similar natural variation in male-killing *Wolbachia*-infection was found in *Drosophila *[27]. Interestingly, the latter found complete absence of infections in some populations despite the absence of resistance mechanisms.

How spatial structure affects the spread of male-killing endosymbionts is poorly documented. In general, imperfect maternal inheritance and direct physiological costs to infection are acknowledged to impede their spread within local populations [3]. Groenenboom & Hogeweg [28] showed that a perfectly transmitted male-killer may invade in a single population with spatial structure (i.e. taking into account neighboring interactions between individuals) without driving the population to extinction. The emerging pattern formation by hosts is here responsible for its persistence under conditions of perfect transmission and fitness compensation. However, because (i) individual interactions in spatially structured insect populations go beyond direct neighbors and (ii) high dispersal rates between populations are necessary for metapopulation dynamics [29], it is doubtful that the local-scale mechanisms presented by [28] can be acknowledged as potential reasons for the presence of high male killer prevalence in natural insect populations.

Dispersal is an important trait within a metapopulation context that is influenced by various selective pressures [30,31]. These comprise avoidance of competition for resources [32], minimizing kin competition [33-37], inbreeding avoidance [38] or coping with temporal variability of resource availability [39-41]. In general, individuals should disperse as long as their (inclusive) fitness expectations are higher outside their natal habitat [42,43]. Consequently, when dispersal costs are higher than expected fitness (e.g., due to high dispersal mortality) dispersal is disfavored (e.g., [31,40]). Dispersal rates therefore increase with increasing environmental stochasticity or external extinction probability and decrease when dispersal costs (dispersal mortality) increase (e.g. [37,43,44]).

Male killing endosymbionts affect host reproduction by relaxing offspring competition (both between kin [5,18,45] but also between non-kin [46]), altering sex ratio [10,45] and subsequent the within-population genetic structure [13]. Because these manipulation are expected to influence the evolution of host dispersal strategies under different conditions of environmental stochasticity and dispersal mortality, we questioned (i) under which of these conditions male killing infections affect the evolution of dispersal rates, (ii) whether and under which environmental conditions male killing endosymbionts get fixated (host extinction) or disappear (curing) and (iii) under which of these condition high among-patch variation in infection prevalence can be retrieved. Our analyses are built on an individual-based model simulating the evolution of dispersal strategies in metapopulations under different levels of environmental stochasticity and dispersal mortality.

## Results

**Figure 1:**
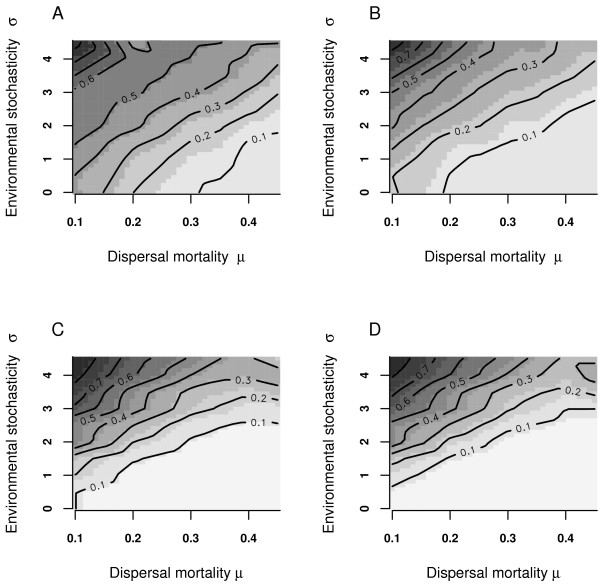
**Dispersal rates of males and females**. Contour plot of average host emigration probability (males, A; females, B) in the metapopulation under infected (no extinction of host and/or endosymbiont) and uninfected conditions(males, C; females, D). The x-axis gives dispersal mortality (*μ*), the y-axis environmental variability (*σ*).

The evolving mean population dispersal probabilities approached equilibrium after less than 2000 generations in all simulation experiments. Similarly, sex ratio, the proportion of infected individuals, and the number of infected populations stabilized after this number of simulation steps. The equilibrium fraction of occupied patches was in all cases, excluding extinct metapopulations, larger than 92% (lowest value 92.4% for *μ *= 0.3, *σ *= 4). In the reference simulation (no endosymbiont invasion), all patches were occupied under all conditions. The invasion of male-killing endosymbionts led to increased dispersal rates (Fig 1), especially under conditions of high *μ *and low *σ*. The increase was pronounced for males (Fig 1A*versus *Fig 1C) compared to females (Fig 1B*versus *Fig 1D).

**Figure 2:**
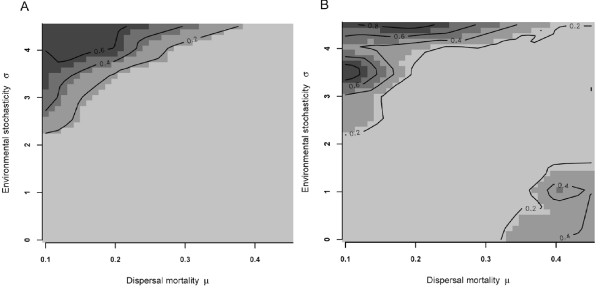
**Metapopulation and endosymbiont extinction**. Effect of endosymbionts invasion on metapopulation extinction probability (A) and the probability that a surviving metapopulation gets infection-free (B).

While extinction did not occur under reference simulations, the invasion of male-killing endosymbionts led to frequent metapopulation extinction under conditions of low *μ *and high *σ *(Fig. 2A). These are conditions that favor high host emigration and thus create more or less a panmictic population structure. Under conditions of high dispersal, metapopulations also occasionally lose endosymbiont infections stochastically and are therefore rescued from eventual extinction. In the case of low dispersal rates, fast deterministic extinction of infected populations may result in the extinction ('curing') of the endosymbiont (Fig. 2B).

**Figure 3:**
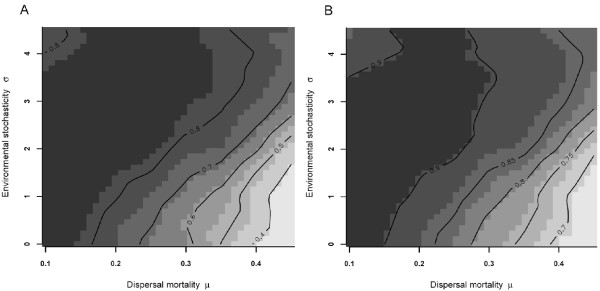
**Infection rates and sex ratio**. Average host infection rates (number of infected females/total population size; A) and sex-ratio (number of females/total population size; B) within host metapopulations under infected conditions (no extinction of host and/or endosymbiont).

**Figure 4:**
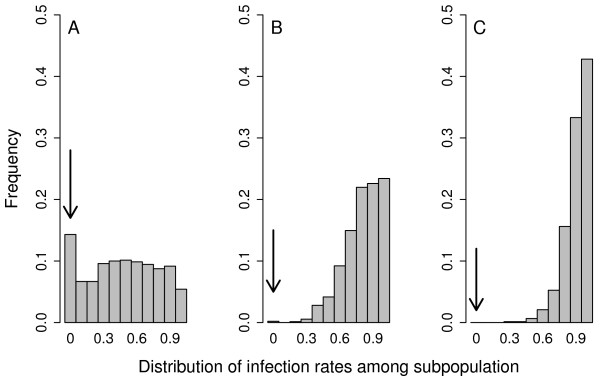
**Among population variation**. Variation in infection rates among local populations in non-extinct metapopulations. Arrows indicate the fraction of non-infected local populations. Histograms (based on 10 replicate simulations) are given for three simulation runs representative of conditions were metapopulations experience high probabilities of losing endosymbionts (A, *μ *= 0.4, *σ *= 0.5), usually show stable coexistence with, on average, high infection rates (B, *μ *= 0.3, *σ *= 2), and the risk of host/endosymbiont metapopulation extinction is high (C, *μ *= 0.1, *σ *= 4).

With more moderate emigration probabilities evolving (*μ*, *σ *both low, or intermediate *μ *and high *σ*), our simulations predict that endosymbionts reach infection rates beyond 80% in non extinct host or endosymbiont metapopulations (Fig 3A). Similarly, sex-ratio's become highly skewed towards females under such conditions (Fig. 3B). Clearly, among population variation in infection-rates (i.e. number of infected females/total population size) within surviving metapopulations is highest under conditions of high *μ *and low *σ *(with a considerable fraction of infection free populations; Fig. 4) and becomes more homogenous with generally high local infection rates in the surviving metapopulations as emigration probabilities go up (low *μ *and high *σ*).

## Discussion

Our simulations show that the invasion of male-killing endosymbionts in a host metapopulation affects the evolution of host dispersal rates. The overall infection rates will depend on the prevailing environmental stochasticity and dispersal mortality. Under conditions supportive for high dispersal in the host population, extinction of the whole host metapopulation, and consequently the parasite population too, become highly probable. In contrast, the probability of endosymbiont extinction increases under conditions that disfavor high dispersal.

Extinction of the host metapopulation under high dispersal rates, which create a panmictic population structure, is similar as for mathematical models that considered single-population dynamics [5,11]. However, the more important results of our simulations is (i) that low dispersal rates may lead to a deterministic extinction (curing) of the endosymbiont and that (ii) high infection rates may not necessarily lead to the extinction of the entire host (meta)population. As previously documented [31], emigration probability increases with decreasing costs of dispersal (*μ*) and increasing environmental variability (*σ*); thus curing occurred in simulations with low *σ *and high *μ*.

Under all conditions, infected populations need male immigration from uninfected populations as male killing rapidly leads to a pure-female population with elevated dispersal rates. Consequently, the extinction probability of infected patches increases with the fraction of infected populations within the metapopulation because fewer and fewer males are produced in the whole metapopulation. This leads to disproportionally increase of the absolute numbers of patches that become extinct over the fraction of infected populations. In contrast, the recolonization of empty patches by infected females only linearly increases with the fraction of infected populations. This eventually leads to a stabilization of the fraction of infected populations, and subsequently the overall infection rates.

**Figure 5:**
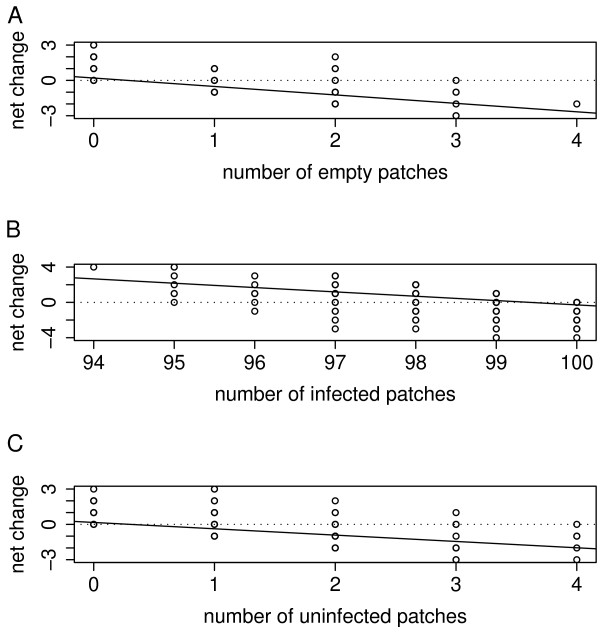
**Feedback mechanisms**. Net changes in the number of empty patches (patch extinction; A), the number of infected patches (B) and the number of uninfected patches (C) in a metapopulation with stable infection rates, plotted over the number of respectively empty, infected and uninfected patches. Regression lines indicate areas where negative feedback regulation occur (*μ *= 0.3, *σ *= 2). Net changes are calculated as the absolute difference in the number of empty (A), infected (B) or uninfected (C) patches at time *t+1 *in relation to the actual number of patches at time *t*.

Stabilization occurs predominantly through the negative feedback on patch extinction, by which the fraction of extinct patches in the next time step *t+1 *decreases when the fraction of extinct patches on a certain moment *t *is larger than 0.04 (Fig. 5A). Interestingly, a similar feedback also emerges for changes in infected (Fig 5B) and uninfected patches (Fig 5C). For uninfected patches and extinct patches, the negative feedback is only prominent when the number of uninfected or extinct populations is low. This implies that when the fraction of uninfected or extinct patches rises above this threshold (this occurs evidently in metapopulations that go extinct or get cured from the infection), stochastic dynamics may induce the curing or extinction of the metapopulation due to positive feedback probabilities. Obviously, this stabilizing mechanism by dispersal is responsible for the smaller range of local infection rates in metapopulations that persist under high dispersal (see Fig 4B, C) compared to conditions with low dispersal (fig 4A). More-over, the higher number of uninfected surviving females (and males evidently) induce a positive feedback on the founding of uninfected populations which on their turn have overall low chances to be colonized by infected females, eventually leading to the curing of the entire metapopulation.

This finding confirms the prediction that frequencies of selfish gene elements (a.o., meiotic drive elements, cytoplasmatic incompatibility, male killers, feminizers; [47]) are a dynamic consequence of local extinction-colonization events in spatially structured population [47]. The only study [28] that explicitly addressed the persistence of male killing endosymbionts and infection prevalence within a spatial setting confirmed the importance of colonization-extinction dynamics, although at the local scale (i.e. within in a population). The resulting pattern formation (i.e., wave patterns by which infections spread quickly leave behind empty space that can only be filled by uninfected individuals) explained male killer persistence, even when transmission efficiency was perfect. In this spatial automata model [28], pattern formation was significantly affected by fitness compensations for survival. In our individual based model, however, no explicit fitness compensations were introduced. Instead, the reduced competition in infected populations fully compensates the fact that infected females lose half of their offspring. This compensation emerges by default and depends on the within-population infection rate. Interestingly, infected females also relax competition between non-kin offspring and consequently strongly influence interdemic (group) selection [46,47].

The induced changes in resource and kin competition by male killing endosymbionts are responsible for the disproportional increase in male emigration compared to that of females. The evolutionary mechanism underlying this sex-specific dispersal [48] is different from the evolution of sex-indifferent dispersal [42,43], but it is evident that it is of particular relevance for the rescue of infected population (Bonte *et al*., submitted for publication). Such populations face the risk of a depletion of males and consequently local extinction in the absence of male immigration.

Our sensitivity analysis with respect to the carrying capacity revealed that the modeling results are robust for larger *K*. Only when K is low, a significant change in extinction and curing probabilities were detected. Evidently, this is due to increased effects of stochasticity in smaller populations [49]. However, since our model reflects endosymbiont invasions in arthropod populations, altered dynamics at low *K *can be disregarded. Besides *K*, male limitation, with subsequent Allee effects on mating, decrease host metapopulation viability when the number of mating events for each male is limited [50,51]. We did not model this implicitly, but models run for monogamous paring systems (compared to the polygynous system described here) indeed confirmed overall low (mostly zero) survival probabilities for invaded host metapopulations (Bonte *et al*., unpub. results).

Evidently, the probability of endosymbionts extinction (the 'curing') is expected to depend on the initial infection rate. Sensitivity analysis showed that high initial infection rates (*I *= 0.5; *I *= 0.8) always led to metapopulation extinction under conditions favoring high dispersal in the host (low *μ *and high *σ*). However, initially high infection rates do not affect the phenomenon of metapopulation curing (only endosymbionts go extinct). In contrast, when initial infection rates were very low and non-evenly distributed only slight increases of parasite extinction rates were observed under conditions that disfavor high dispersal. The fate of male-killer infections also strongly depends on the local population size (*K*). When *K *was very low (*K *= 10), male killers always disappeared from the metapopulations. Entire metapopulations got extinct under environmental conditions that disfavored dispersal (*μ *> 3, *σ *< 2). Under conditions of low *μ *and high *σ*, metapopulation extinction rates always exceeded 0.56, because the remaining fraction got entirely cured by stochastic processes. In the latter, dispersal rates increased up to 15–25% due to the increasing importance of kin competition [37]. At the other extreme, when *K *was increased (K = 250, 500), only a slight decrease in overall extinction (respectively 0.07 (*K *= 250), 0.06 (*K *= 500), compared to 0.09 for *K = 100*) and curing rates were observed (respectively 0.16 (*K *= 250), 0.14 (*K *= 500), compared to 0.18 for *K = 100*), obviously due to an increase in population size. Because insect populations are expected to occur at high local population densities, we assume our modeling results therefore to be reliable with respect to the envisioned biological system.

As demonstrated by our simulations, endosymbionts are only able to persist under intermediate levels of host dispersal. Even exceptional infection rates of up to 90% and associated skewed sex ratio's, may be stable under conditions that are characterized by low environmental stochasticity and low dispersal costs. Such stability does not require behavioral changes in mating system or fitness costs for infected individuals. For example, low environmental variation and low dispersal costs for butterflies in tropical forests [20,52] could explain the high infection rates reported for these species. Accordingly, agrobiont species (experiencing high dispersal costs after reproduction in contemporary landscapes; e.g. [53]) show, on average, low to intermediate infection rates [20]. These observations are thus in good agreement with our result that the spatial dynamics in host metapopulations can be important for the establishment of infection rates by male-killing endosymbionts. Our simulations also showed that strong among-population variation in infection rates may occur under ecological conditions that support the evolution of low to intermediate evolutionary stable dispersal rates in hosts. Relating recently observed among-population heterogeneity [24,26] in local infection rates to the spatial structure and environmental conditions of the entire metapopulation could consequently provide a more quantitative validation of our hypothesis. Our simulation experiments therefore add to recent theoretical work [28,50,51,54] that highlights the crucial importance of spatial ecological dynamics for evolutionary host-parasite processes.

## Conclusion

The invasion of male killer endosymbionts is responsible for the evolution towards higher dispersal rates in their host. The resulting sex-specific dispersal rates in host metapopulations that are invaded by male-killing endosymbionts strongly determine the level of infection rates and related host-endosymbiont population dynamics. The influence of environmental conditions on host dispersal allows for the emergence of high but stable infection rates under a wide range of environmental conditions, which favor the evolution of intermediate host dispersal. In contrast, endosymbionts are predicted to carry high extinction risks under either low or high host dispersal activities. Under high dispersal, this is either due to fixation of the infection (and extinction of the host metapopulation) or due to accidental loss of the infection from host metapopulations at the brink of global extinction, which may, however, recover after the infection is lost. In contrast, low dispersal rates may lead to deterministic curing of the host population.

## Methods

### The model

#### The landscape

For our simulation experiments we used an extended version of an individual-based model of insect dispersal in patchy landscapes of 100 habitat patches (*n*) with carrying capacities *K *[15-17]. Patch capacity was set to *K *= 100 individuals.

#### The individual

Each individual is characterized by its sex, its affiliation with a specific patch (*i*), by four alleles at two different diploid loci that determine male (*d*_*m*_), respectively female (*d*_*f*_) dispersal propensity. The allele values were initially randomly drawn from a uniform distribution [0–1]. Further, individuals are characterized by their infection status (infected versus uninfected) which they solely inherit from their mother. In our model, individuals simultaneously disperse before mating and production of offspring; each individual has only one opportunity to disperse. Dispersing individuals die with a probability *μ *(dispersal mortality), regardless of patch origin.

#### Population dynamics

Local population dynamics are governed by density-dependent reproduction of individuals. After mating with a randomly drawn local male (thus allowing polygamy), a female gives birth to Λ offspring, where Λ is a Poisson-distributed number with a patch- and time-specific mean, Λ_mean_(*t*, patch). For each generation, the mean value of Λ_mean_(*t*, patch) is drawn from a lognormal distribution with mean *λ *and a standard deviation *σ*. In our simulations, *λ *was set to 4, a value typical for arthropod demography. *σ *subsequently determines the degree of environmental fluctuations which are assumed to be uncorrelated in space and time. Offspring are randomly assigned to the male or female sex, but male offspring from infected females die immediately after conception. Remaining offspring develop into mature individuals with a density-dependent survival probability *s*:

(1) $$\begin{array}{l} s=\frac{1}{\left(1+aN_{i}\right)}\\ \text{with }a=\frac{\lambda -1}{K} \end{array}$$

Here *N*_*i *_represents the expected population size in patch *i. K is *the carrying capacity of patch *i *(identical for all patches). This means that there is no fitness benefit for infected females, but for groups with infected females, population growth increases as female offspring are released from competition with males.

#### Dispersal

After all individuals have reached maturity, they disperse according to their genetically determined dispersal probability *d *(i.e., according to mean value of their sex-specific dispersal allele, *d*_*m *_or *d*_*f*_). The dispersal alleles were freely recombined during reproduction. We assume global dispersal; that is, a successful disperser reaches any patch in the landscape (except its home patch) with the same probability (1-*μ*)/(*n-1*). Dispersal probability was sex-specific and unconditional, i.e. assuming dispersing arthropods taking their decision without taking into account any information from the patch. Dispersal alleles were allowed to change by mutation, thus allowing for the evolution of sex-specific dispersal strategies. We implemented sex-specific dispersal because we expect male-killing endosymbionts to affect both local demography and sex-ratio, thereby potentially inducing different 'games' for males and females.

#### Mutation rate and stochasticity

To promote greater variability of genotypes in the first generations and to reduce the influence of mutations on the stability of the final result, we let mutation rates exponentially decrease from ~0.1 to < 0.001 over the course of the simulation experiments (5000 generations; see e.g. [16]). A mutation comprises a shift towards a new random value from the initial uniform [0–1] distribution. No external catastrophes were simulated; instead we allowed demographic stochasticity and environmentally caused fluctuations (0 ≤ *σ *≤ 5) in offspring number (Λ).

#### Simulation experiment

We ran scenarios to test whether the presence of infections in the metapopulation influenced the dispersal rate of hosts, the metapopulation extinction probability and the eventual rate of infection. Therefore, simulations were run either without infections (*I *= 0) or with an initial female infection rate *I *= 0.10 randomly distributed over patches. Simulations for both scenarios were replicated (n = 100) for dispersal mortality and environmental stochasticity (Table 1). Because low or high initial infection rates allow for high rates of respectively curing or metapopulation extinction just by demographic fluctuations, we ran sensitivity analyses for relevant ranges of *I *and *K *(Table 1).

**Table 1:** Parameters of the model

Parameter	Description	Ranges tested
***model analysis***		
*K*	Carrying capacity local populations	100
*I*	Initial infection rates	0, 0.1
*λ*	mean offspring number	4
*σ*	standard deviation in mean offspring number; reflects environmental stochasticity	0, 0.5,...,4.5
*μ*	dispersal mortality	0.1, 0.15...,0.45
***Sensitivity analysis***		
*K*	Carrying capacity	10, 250, 500
*I*	Initial infection rates	0.001, 0.01, 0.02, 0.50, 0.80

Host infection rates were calculated as the number of infected females/total population size, sex-ratio as the number of females/total population size. Metapopulation extinction probability was calculated as the number of simulation runs with metapopulation extinctions divided by the total number of replicates for the respective scenario. Other metapopulation parameters were only estimated for the surviving ones.

## Authors' contributions

The work presented here was carried out in collaboration between all authors. DB conceptualized the research questions, implemented the model, analyzed the data, interpreted the results and wrote the paper. TH and HJP designed an earlier version of the model, discussed analyses, interpretation, and presentation. All authors have contributed to, seen and approved the manuscript.
